# Fructose Removal from the Diet Reverses Inflammation, Mitochondrial Dysfunction, and Oxidative Stress in Hippocampus

**DOI:** 10.3390/antiox10030487

**Published:** 2021-03-20

**Authors:** Arianna Mazzoli, Maria Stefania Spagnuolo, Martina Nazzaro, Cristina Gatto, Susanna Iossa, Luisa Cigliano

**Affiliations:** 1 Department of Biology, University of Naples Federico II, Complesso Universitario Monte Sant’Angelo, 80126 Naples, Italy; arianna.mazzoli@unina.it (A.M.); martina.nazzaro@unina.it (M.N.); cristina.gatto@unina.it (C.G.); susiossa@unina.it (S.I.); 2 Department of Bio-Agrofood Science, Institute for the Animal Production System, National Research Council, 80147 Naples, Italy; mariastefania.spagnuolo@cnr.it

**Keywords:** hippocampus, mitochondria, fructose diet, young rat, inflammation, oxidative stress, haptoglobin, neurofilament-M, PSD-95

## Abstract

Young age is often characterized by high consumption of processed foods and fruit juices rich in fructose, which, besides inducing a tendency to become overweight, can promote alterations in brain function. The aim of this study was therefore to (a) clarify brain effects resulting from fructose consumption in juvenile age, a critical phase for brain development, and (b) verify whether these alterations can be rescued after removing fructose from the diet. Young rats were fed a fructose-rich or control diet for 3 weeks. Fructose-fed rats were then fed a control diet for a further 3 weeks. We evaluated mitochondrial bioenergetics by high-resolution respirometry in the hippocampus, a brain area that is critically involved in learning and memory. Glucose transporter-5, fructose and uric acid levels, oxidative status, and inflammatory and synaptic markers were investigated by Western blotting and spectrophotometric or enzyme-linked immunosorbent assays. A short-term fructose-rich diet induced mitochondrial dysfunction and oxidative stress, associated with an increased concentration of inflammatory markers and decreased Neurofilament-M and post-synaptic density protein 95. These alterations, except for increases in haptoglobin and nitrotyrosine, were recovered by returning to a control diet. Overall, our results point to the dangerous effects of excessive consumption of fructose in young age but also highlight the effect of partial recovery by switching back to a control diet.

## 1. Introduction

In previous decades, a significant increase in the fructose content of the human diet has occurred, far above what is introduced daily with fruits and vegetables, because of the increased consumption of industrial foods and the extensive commercial use of high-fructose corn syrup (HFCS) as a sweetener for beverages, coffee, snacks, and bakery foods [1,2]. High fructose consumption has long been known to expose the consumer to metabolic health risks, such as obesity, lipid alterations, insulin resistance, and inflammation [3,4,5,6].

Recently, different lines of evidence in animal models highlighted the marked effects of this sugar on brain structure and function [7,8]. In fact, long-term feeding with fructose can cause brain alterations such as insulin signaling [9], neurogenesis [10], and redox homeostasis, as well as neuroinflammation [11,12]. Further experimental studies have highlighted possible mechanisms at the basis of cognitive deficits induced by the consumption of this sugar [13,14,15]. We also reported that a short-term fructose diet induced neuroinflammation, oxidative stress, and impairment of insulin signaling in the hippocampi of young and adult rats [16]. It has been shown that just a week of fructose ingestion negatively impacts brain plasticity [17]. Interestingly, a very recent study showed that the spatial memory deficit, neuroinflammation, and brain protein changes induced by 12 weeks of fructose drinking in adult rats persisted for one month after its elimination from the diet [18].

Young people are among the highest consumers of sweet foods, especially fructose-sweetened beverages [19]. Metabolic risk factors related to poor nutrition can arise at a very early age; hence, investigations are required to clarify the brain consequences resulting from a diet rich in fructose at a critical stage of development. Indeed, despite the importance of this issue, few studies have been performed in rodents, providing evidence that, in childhood and adolescence, critical periods of neurocognitive development, the impact of high dietary fructose consumption on hippocampal function is particularly damaging [16,20]. Of note, a very recent study of neuroimaging on 103 healthy children aged 7 to 11 years supported animal data and translated these findings to humans, providing evidence that increases in dietary fructose are associated with alterations in hippocampal structure and connectivity [21].

Given the limited research on the persistence of the brain alterations caused by high fructose consumption in individuals of juvenile age, in this study we focused our analysis on the hippocampi of young (30 days old) rats fed a fructose-rich (F) or control diet (C) for 3 weeks. After this period, fructose-fed rats were fed a control diet for a further 3 weeks (FR) and compared with rats that received the control diet for the entire period (CR) in order to clarify whether alterations induced by a short-term fructose diet can be rescued after removing fructose from the diet. Attention was focused on the hippocampus, a brain area responsible for learning, memory, and food intake regulation that is particularly susceptible to dietary and metabolic insults [20,22,23]. This investigation aimed to highlight the early effects (3 weeks of treatment) of the fructose diet on hippocampal expression of glucose transporter-5 (Glut-5), fructose and uric acid levels, inflammation and redox homeostasis in terms of mitochondrial function, and oxidative damage to lipids and proteins as well as antioxidant enzyme activities. In addition, as alterations to redox homeostasis are closely connected with changes in neuronal function, the effects of dietary fructose treatment on neurofilament-M (NF-M), synaptophysin, synaptotagmin, and post-synaptic density protein 95 (PSD 95) were also investigated.

## 2. Materials and Methods

### 2.1. Materials

Goat anti-Rabbit-Horseradish peroxidase-conjugated IgGs (GAR-HRP) and Goat anti-Mouse-Horseradish peroxidase-conjugated IgGs (GAM-HRP) were purchased from Immunoreagent (Raleigh, NC, USA). Fuji Super RX 100 films (Laboratorio Elettronico Di Precisione, Naples, Italy), polyvinylidene difluoride (PVDF) membrane (GE Healthcare, Milan, Italy), and dye reagent for protein titration (Bio-Rad, Hercules, CA, USA) were used for Western blotting. Salts, buffers, and Bovine serum albumin fraction V (BSA) were purchased from Sigma-Aldrich (St. Louis, MO, USA).

### 2.2. Experimental Design

Male Wistar rats (Charles River, Italy), 30 days of age, were purchased from Charles River (Calco, Como, Italy) and housed as previously described [16]. Treatment, management, and euthanasia of animals met the guidelines set out by the Italian Health Ministry, and all experimental procedures were approved by “Comitato Etico-Scientifico per la Sperimentazione Animale” of the University of Naples Federico II (448/2019-PR).

The rats were divided into two groups, one fed a fructose rich diet (F group) and the other fed a control diet (C group) for 3 weeks. The control and fructose-rich diet compositions are reported in Table 1. At the end of the 3-week period, half of the rats from each group were euthanized and the other half were maintained on a control diet (FR and CR groups) for a further 3 weeks. Body weight and food and water intake were monitored daily. At the end of the experimental period, the animals were euthanized and decapitated. The hippocampi were harvested and dissected [24]. Mitochondrial oxygen consumption was immediately assessed in little sections of tissue. Pieces of each sample were fixed for immunofluorescence analysis, and the remaining samples were snap frozen in liquid nitrogen and stored at −80 °C.

### 2.3. Metabolic Parameters

Colorimetric enzymatic methods were used to assess the contents of fructose and uric acid in the hippocampus using commercial kits (Sigma Aldrich, St. Louis, MO, USA for fructose, and GS Diagnostics SRL, Guidonia Montecelio, Rome, Italy for uric acid).

### 2.4. Protein Extraction

Aliquots of frozen hippocampus (about 80 mg) were homogenized in six volumes (*w*/*v*) of cold RIPA buffer, as previously described [25,26]. The protein concentration was measured with colorimetric Bio-Rad Bradford Protein Assays using a commercial kit (Bio-Rad, Hercules, CA, USA) according to the manufacturer’s instructions. Selected markers of inflammation (TNF-alpha; nuclear factor kappa-light-chain-enhancer of activated B cells, NF-kB; ionized calcium binding adapter 1, Iba 1; glial fibrillary acidic protein, GFAP; Haptoglobin, Hpt), synaptic function (synaptophysin, synaptotagmin I, post synaptic density protein 95, PSD 95), and protein oxidative damage (Nitro-Tyrosine) were then evaluated by Western blotting or ELISA, as described below.

### 2.5. Analysis of Tumor Necrosis Factor Alpha (TNF-Alpha)

The TNF-alpha Duo-Set kit (R&D, DBA Italia) was used to measure the TNF-alpha concentration by sandwich ELISA, as previously described [27]. Data are expressed in pg of TNF-alpha per mg of proteins (assessed by Bradford Protein assay).

### 2.6. Western Blotting

Aliquots (30 µg of protein) of hippocampus were analyzed by electrophoresis in denaturing and reducing conditions [28] on 12.5% (to quantify Glut-5, Iba 1, GFAP, synaptophysin, adiponectin) or 10% (NF-kB, PSD 95, synaptotagmin I) polyacrylamide gel. Proteins were transferred onto PVDF membrane [29], which was then washed by T-TBS (130 mM NaCl, 20 mM Tris-HCl, 0.05% Tween 20, pH 7.4). The membrane was incubated (60 min, 37 °C) in T-TBS containing 5% non-fat milk or 3% BSA (blocking step), and then the following detections were performed: Glut-5 by rabbit anti Glut-5 IgG (Invitrogen, Carlsbad, CA, USA, 0.5 μg/mL in T-TBS containing 3% BSA; overnight, 4 °C), followed by GAR-HRP IgG (1:45,000 dilution; 1 h, 37 °C); adiponectin by rabbit anti-human adiponectin IgG (Immunological Sciences, Rome, Italy; 1:500 dilution in in T-TBS containing 3% BSA; overnight, 4 °C), followed by GAR-HRP IgG (1:15,000 dilution; 1 h, 37 °C); peroxisome proliferator-activated receptor gamma coactivator 1-alpha (PGC-1α) by rabbit anti-human PGC-1 IgG (Millipore, Burlington, MA, USA; 1:2000 in in T-TBS containing 3% BSA; overnight, 4 °C) with), followed by GAR-HRP IgG (1:20,000 dilution; 1 h, 37 °C); p-NF-kB and NF-kB by monoclonal mouse antibodies (Santa Cruz biotechnology, Dallas, TX, USA; 1:200 and 1:500, respectively, in 3% BSA; overnight, 4 °C), followed by GAM-HRP IgG (1:50,000 and 1:15,000, respectively, in 3% BSA; 1 h, 37 °C); Iba 1 by anti-Iba1 (1022-5) IgG (Santa Cruz biotechnology; 1: 500 in 3% BSA; overnight, 4 °C) and then GAM-HRP (1:10,000 in 1% non-fat milk; 1 h, 37 °C); GFAP by rabbit anti-human GFAP IgG (Cell Signaling, Beverly, MA, USA; 1:1000 in 1% non-fat milk; overnight, 4 °C), followed by GAR-HRP IgG (1:150,000 in 5% non-fat milk; 1 h, 37 °C); synaptophysin by anti-synaptophysin IgG (Merk Millipore, Milan, Italy; 1:150,000 in 3% BSA; overnight, 4 °C), followed by GAR-HRP IgG (1:20,000 dilution in 1% non-fat milk; 1 h, 37 °C); synaptotagmin I by anti-synaptotagmin I IgG (Cell Signaling; 1:1000 in 3% non-fat milk; overnight, 4 °C) followed by GAR-HRP IgG (1:100,000 dilution in 3% non-fat milk; 1 h, 37 °C); and PSD 95 by anti-PSD 95 IgG (Cell Signaling; 1:1000 in 3% non-fat milk; overnight, 4 °C), followed by GAR-HRP IgG (1:70,000 dilution in 3% non-fat milk; 1 h, 37 °C).

To load the control, β-actin was revealed after the detection of each marker. For this aim, the membranes were stripped [27] and then treated with mouse anti-β-actin IgG (1000 in 0.25% non-fat milk; overnight, 4 °C), followed by GAM-HRP IgG (1:30,000 in 0.25% non-fat milk; 1 h, 37 °C). The Excellent Chemiluminescent detection kit (Cyanagen s.r.l., Bologna, Italy) was used for detection. Chemidoc or digital images of X-ray films exposed to immunostained membranes were used for the densitometric analysis, and quantification was carried out with Un-Scan-It gel software (Silk Scientific, Orem, UT, USA).

### 2.7. Haptoglobin (Hpt) and Hemoglobin (Hb) Evaluation

Hpt concentration in hippocampus samples was measured by ELISA using rabbit anti-human haptoglobin (Sigma-Aldrich, St. Louis, MO, USA) in accordance with Mazzoli et al., 2020 [30].

The Hb concentration was measured by ELISA in hippocampus samples (diluted 1:200, 1:800, 1:1500) using rabbit anti-human Hb IgG (1: 500 dilution in T-TBS containing 0.25% BSA; Sigma-Aldrich, St. Louis, MO, USA), followed by 60 µL of GAR-HRP IgG (1:5000 dilution, 1 h, 37 °C). Coating, washing, and blocking were carried out as previously reported [31].

### 2.8. Mitochondrial Analyses in Rat Hippocampi

Hippocampus samples were homogenized (1:1000, *w*/*v*) in Mir05 medium (110 mM sucrose, 60 mM K-lactobionate, 20 mM Hepes, 20 mM taurine, 10 mM KH2PO4, 6 mM MgCl_2_, 0.5 mM EGTA, 0.1% fatty acid free BSA, pH 7.0).

Aliquots of homogenates (2 mg) were used to measure the oxygen flux (pmol O2 s^−1^ mL^−1^) with an Oxygraph-2k (O2k, OROBOROS INSTRUMENTS, Innsbruck, Austria) at 37 °C.

A substrate, uncoupler, inhibitor titration (SUIT) protocol was applied to assess qualitative and quantitative mitochondrial changes [32]. Leak respiration (CI_L_) through complex I (CI) was evaluated using malate (0.5 mM), pyruvate (5 mM), and glutamate (10 mM). Phosphorylating respiration supported by complex I (CI_P_) was assessed by the addition of 2.5 mM ADP. Respiration supported by complexes I and II (C_I&IIP_) was measured by adding 10 mM of succinate. Oligomycin was added at a concentration of 2.5 mM to assess leak respiration (C_I&IIL_). The maximum capacity of the electron transport chain (C_I&IIE_) was obtained by the addition of the uncoupler carbonyl cyanide p-trifluoromethoxyphenylhydrazone (FCCP, 0.5 mM). Rotenone (0.5 μM) was added to determine the maximal capacity supported by CII alone d (C_IIE_). The residual oxygen consumption was established by the addition of the inhibitor antimycin A (2.5 mM), and the resulting value was subtracted from the flux in each run to correct for nonmitochondrial respiration.

Calculation of Intrinsic Mitochondrial Function By using high resolution respirometry (HRR), we were able to assess different respiratory states within the same sample [33,34,35]. From these respiratory states, flux control ratios (FCR) could be calculated for leak respiration (FCR_L_) with electron provision from complex I or complexes I and II and phosphorylating respiration (FCR_P_) with electron provision from complex I or complexes I and II by using the formulas detailed below:

FCR_LI_ = CI_L_/C_I&IIE;_
FCR_LI&II_ = C_I&IIL_/C_I&IIE;_
FCR_PI_ = CI_P_/C_I&IIE_;
FCR_PI&II_ = C_I&IIP_/C_I&IIE._
In addition, flux control factors (FCFs) were calculated as follows:
Coupling efficiency of oxidative phosphorylation = 1 − C_I&IIL_/C_I&IIP_;
Excess capacity of the electron transport chain = 1 − C_I&IIP_/C_I&IIE_;
FCF Complex I = 1 − (C_IIE_/C_I &IIE_);
FCF Complex II = 1 − (CI_P_/C_I + IIP_).

Procedures to test mitochondrial integrity were routinely carried out at the beginning of each measurement by measuring the effect of 10 mM of exogenous cytochrome c on complex I-supported mitochondrial respiration.

### 2.9. Oxidative Stress Markers and Antioxidant Enzymes

Nitro-tyrosine (N-Tyr) titration was carried out by ELISA in hippocampal homogenates (diluted 1:1500, 1:3000, 1:6000) using rabbit anti-N-Tyr IgG (Covalab, distributed by VinciBiochem, Vinci, Italy; 1:600 dilution in T-TBS containing 0.25% BSA), followed by 60 µL of GAR-HRP (1:3500 dilution; 1 h, 37 °C), essentially as previously described [24,36]. Data are reported as OD per mg of proteins.

The extent of lipid peroxidation was determined by measuring thiobarbituric reactive substances (TBARS) using the thiobarbituric acid assay, essentially as previously described [37]. The concentration of TBARS was calculated using a molar extinction coefficient of 1.56 × 10^5^/M/cm and expressed as nmol TBARS per g tissue.

In order to assess superoxide dismutase activity (SOD), little pieces of hippocampus were homogenized in a buffer containing 0.1 mM EDTA, 50 mM KH_2_PO_4_ pH 7.8, 20 mM cythocrome c, 0.1 mM xanthine, and 0.01 units of xanthine oxidase. SOD activity was measured by monitoring, spectrophotometrically (550 nm; 25 °C), the decrease in the reduction rate of cytochrome c by superoxide radicals generated by the xanthine-xanthine oxidase system. One unit of SOD activity is defined as the concentration of enzyme that inhibits cytochrome c reduction by 50% in the presence of xanthine + xanthine oxidase [38].

Glutathione reductase (GR) activity was measured in accordance with a previous report [39]. For the determination of GR activity, the decrease of NADPH absorbance at 340 nm was measured at 30 °C in homogenates from the hippocampus. The reaction mixture contained 0.2 M potassium phosphate buffer, 2 mM EDTA, 2 mM NADPH (in 10 mM Tris-HCl, pH 7), and 20 mM oxidized glutathione (GSSG). The activity was calculated using the NADPH molar extinction coefficient, 6.22 × 10^−5^, considering that one unit of glutathione reductase is defined the amount of enzyme that catalyzes the reduction of 1 μmol of NADPH per minute. The specific activity is expressed in mU per g of tissue.

### 2.10. Immunofluorescence Analysis

Paraffin-embedded sections of hippocampus from all groups were stained with the specific monoclonal antibody against the phosphorylated form of neurofilament-M (p-NF-M, Santa Cruz Biotechnology, Dallas, TX, USA), and slides were stained with DAPI (Sigma Aldrich, Saint Louis, MO, USA). For the analysis, images were acquired with ×40 magnification. Three random fields/section per rat were analyzed using ImageJ (National Institutes of Health, Bethesda, MD, USA). Images were captured and visualized using a Nikon Eclipse E1000 microscope.

### 2.11. Statistical Analysis

Data are reported as mean values ± SEM. The program GraphPad Prism 6 (GraphPad Software, San Diego, CA, USA) was used to verify that raw data had a normal distribution and to perform a one-way ANOVA followed by Tukey’s post-test. *p* < 0.05 was considered significant in the reported analyses.

## 3. Results

### 3.1. Glut-5 Expression, Fructose and Uric Acid Level in Hippocampus

**Figure 1:**
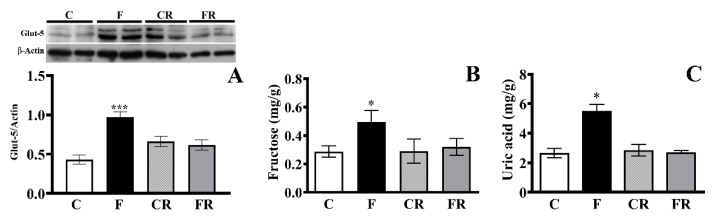
Hippocampus fructose handling. (**A**) Glut-5 level (with representative Western blot), (**B**) fructose and (**C**) uric acid concentrations in the hippocampi of control (C), fructose-fed (F), control rescued (CR), and fructose-rescued (FR) rats. Data are the means ± SEM of eight rats/group. * *p* < 0.05 versus control rats; *** *p* < 0.001 versus control rats. Source of variation: one-way ANOVA followed by Tukey’s post-test.

To obtain information on the delivery of fructose to brain cells, we quantified the protein expression of Glut-5, the specific fructose transporter, as well as the levels of fructose and uric acid, one of the main products of fructose metabolism, in the hippocampus after 3 weeks of consuming a fructose-rich diet. The Glut-5 concentration was significantly higher (*p* < 0.001) in the hippocampi of F rats compared to in C rats, while this increase disappeared in FR rats (Figure 1A). In line with this result, significant increases in hippocampus levels of fructose (*p* < 0.05) and uric acid (*p* < 0.05) were found in F rats compared to C rats, while no significant differences were found in FR rats (Figure 1B,C).

### 3.2. Markers of Hippocampal Inflammation

**Figure 2:**
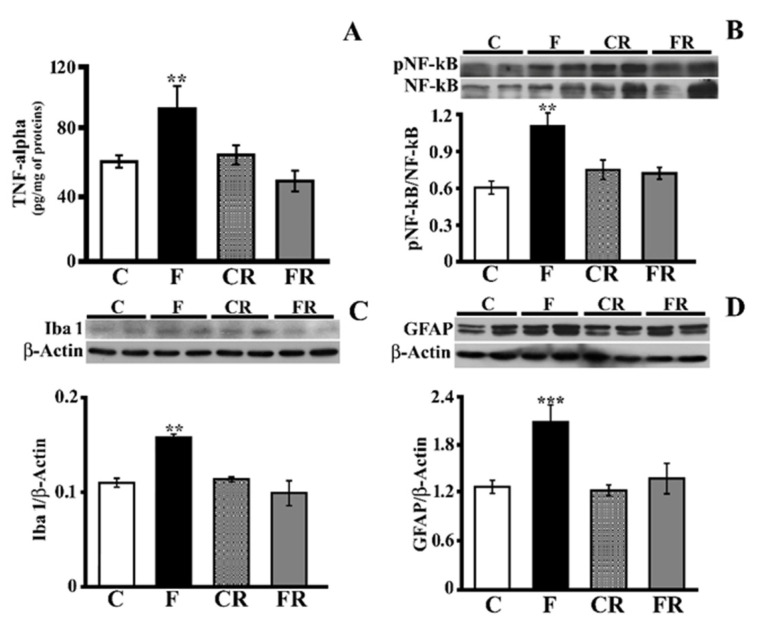
Inflammatory status in the hippocampus (**A**)TNF-alpha concentration (titrated by sandwich ELISA), (**B**) activation of NF-kB (expressed as the pNF-kB/NF-kB ratio with representative Western blot), (**C**) Iba1 level (with representative Western blot), (**D**) GFAP level (with representative Western blot) in protein extracts from the hippocampi of control (C), fructose-fed (F), control rescued (CR), and fructose-rescued (FR) rats. Data are the means ± SEM of eight rats/group. ** *p* < 0.01 versus control rats; *** *p* < 0.001 versus control rats. Source of variation: one-way ANOVA followed by Tukey’s post-test.

We previously reported that short-term fructose feeding was associated with an increase in inflammatory markers (TNF-alpha and GFAP) in the hippocampi of young and adult rats [16]. To further clarify the brain inflammatory mechanisms activated by a fructose-rich diet and the putative ability of a subsequent 3-week control diet to rescue the fructose-induced alterations, we evaluated TNF-alpha, NF-kB activation, GFAP as marker of astrogliosis, and Iba1 as a marker of microglial activation. The TNF-alpha level was significantly higher in the hippocampi of F rats (*p* < 0.01; Figure 2A), while it returned to control levels in FR rats (Figure 2A). In agreement with results from TNF-alpha titration, in the hippocampi of F rats, we found a significant increase in the degree of NF-kB phosphorylation (*p* < 0.01), which returned to levels comparable to those of C rats in FR rats (Figure 2B). Fructose feeding was also associated with increases in Iba1 (*p* < 0.01; Figure 2C) and GFAP (*p* < 0.001; Figure 2D). After switching to the control diet, the above differences disappeared. Overall, these results show that a fructose-rich diet is associated with an increase in key inflammation markers in the hippocampus, and this nutritional insult is fully rescued after switching to a control diet.

**Figure 3:**
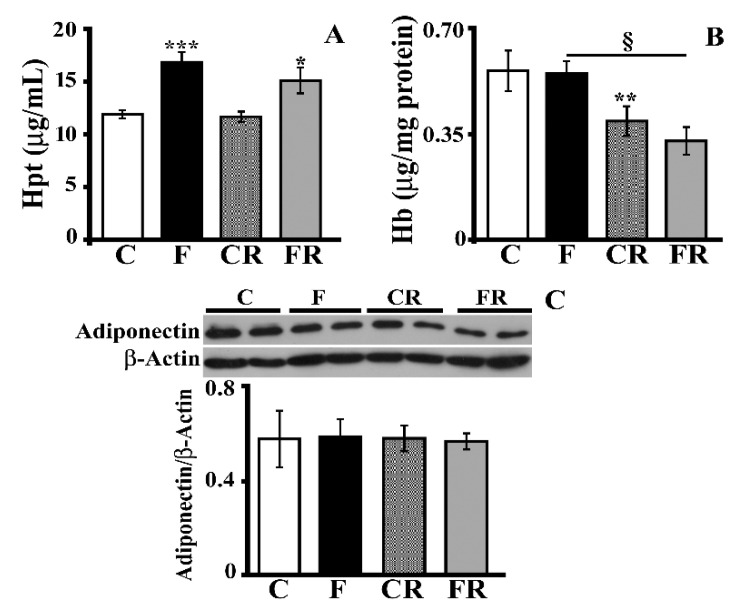
Haptoglobin (Hpt), hemoglobin (Hb), and adiponectin. (**A**) Hpt and (**B**) Hb levels (titrated by ELISA) and (**C**) adiponectin concentration (with representative Western blot) in protein extracts from the hippocampi of control (C), fructose-fed (F), control rescued (CR), and fructose-rescued (FR) rats. Data are the means ± SEM of eight rats/group. * *p* < 0.05; ** *p* < 0.001; *** *p* < 0.001 versus control rats; § *p* < 0.05 versus fructose rats. Source of variation: one-way ANOVA followed by Tukey’s post-test.

Interestingly, this result was not true when the level of hippocampal Hpt, a marker of inflammation [29,40] that is very sensitive to nutritional changes [30,36], was measured (Figure 3A). Indeed, the Hpt level was greater (*p* < 0.001) in F rats with values that also remained significantly higher in FR rats compared with C rats. The increase in Hpt might represent a compensative response to a diet-induced change in Hb, as Hpt is an acute phase protein that binds to free Hb and neutralizes its pro-oxidative action, thus limiting oxidative stress [31]. This hypothesis was excluded by the finding of no alteration in the level of Hb after consumption of a fructose-rich diet (Figure 3B). Of note, the Hb level was decreased both in CR rats compared to C rats (*p* < 0.01) and in FR compared to F rats (*p* < 0.05), thus suggesting an age-dependent effect. No diet-associated variation in adiponectin, an adipokine with a neuroprotective effect [41], was detected in the hippocampi (Figure 3C).

### 3.3. Electron Transport Chain Pathway

Mitochondrial activity was assessed by evaluating the integrated pathway of the electron transport chain. HRR was used to determine the FCR in the hippocampus using a SUIT protocol.

**Figure 4:**
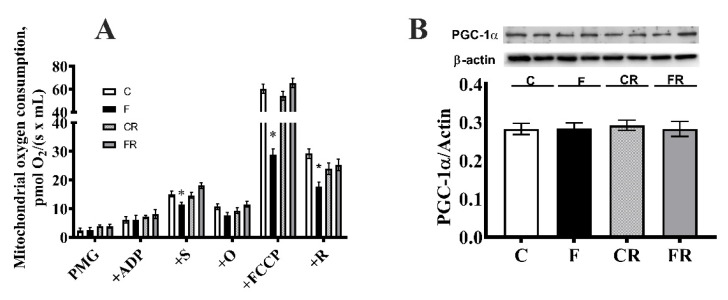
Mitochondrial function. (**A**) Non-normalized respiration after the addition of pyruvate + malate+ glutamate (PMG), ADP, succinate (S), oligomycin (O), FCCP, and rotenone (R), (**B**) PGC-1alpha concentration (with representative Western blot) in the hippocampi from control (C), fructose-fed (F), control-rescued (CR), and fructose-rescued (FR) rats. Values are the means ± SEM of eigth different rats. * *p* < 0.05, compared to C rats (One-way ANOVA followed by Tukey’s post-test). Data are the means ± SEM of eight different rats. * *p* < 0.05 compared to C rats. Source of variation: one-way ANOVA followed by Tukey’s post-test. FCCP: carbonyl cyanide p-trifluoromethoxyphenylhydrazone.

As for mitochondrial oxygen consumption rates, we found a significant decrease in ADP-supported respiration with complex I- and II-linked substrates and in FCCP-stimulated respiration with complex II or complex I and II-linked substrates in F rats (Figure 4A). The analysis of PGC-1α, a marker of mitogenesis, showed no evidence of differences between the rat groups (Figure 4B).

**Figure 5:**
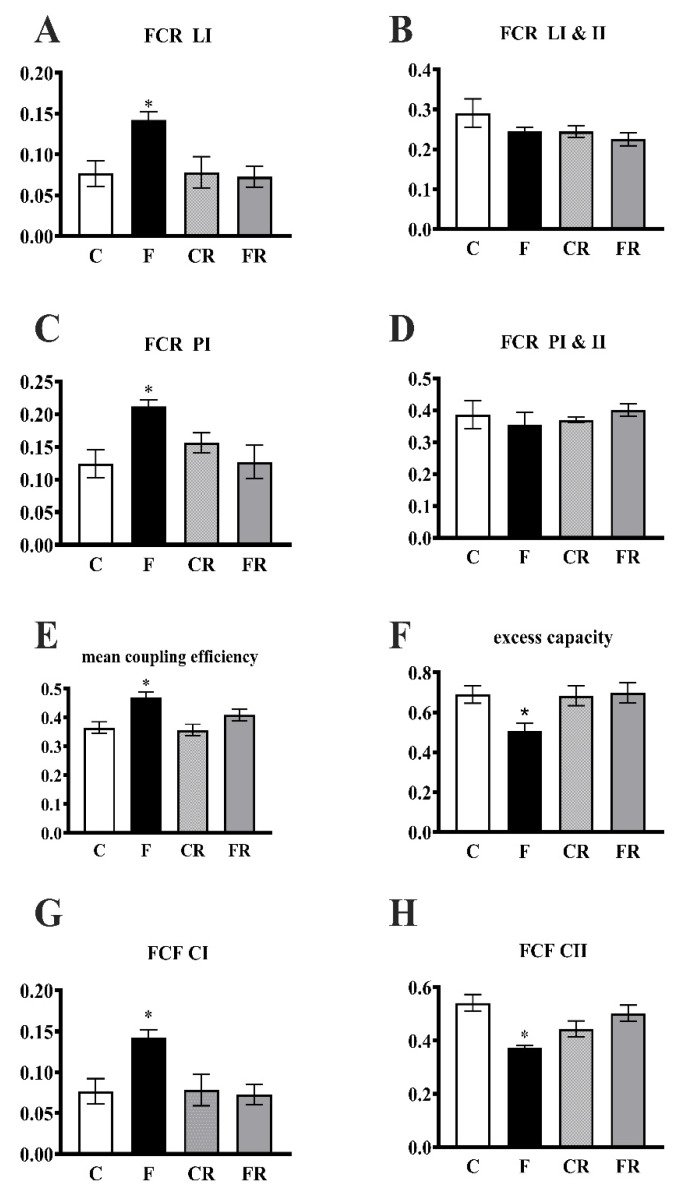
Integrated pathway of the electron transport chain in the hippocampus. Leak respiration with electron provision from complex I (FCR LI) (CIL)/(CI&IIE) (**A**) and complexes I and II (FCR L CI&II (CI&IIL/CI&IIE) (**B**), phosphorylating respiration with electron provision from complex I (FCR PI) (CIP/CI&IIE) (**C**) and complexes I and II (FCR PI&II) (CI&IIP/CI&IIE) (**D**), coupling efficiency of oxidative phosphorylation (1—CI&IIL/CI&IIP) (**E**), apparent excess capacity of the electron transport chain (1—CI&IIP/CI&IIE) (**F**), flux control factor (FCF) for complex I (**G**) and complex II (**H**) in the hippocampi from control (C), fructose-fed (F), control-rescued (CR), and fructose-rescued (FR) rats. Values are the means ± SEM of eight different rats. * *p* < 0.05 compared to C rats. Source of variation: one-way ANOVA followed by Tukey’s post-test. CIL = leak respiration with complex I substrate; CI&IIL = leak respiration with complex I and II substrates; CIP = phosphorylating respiration with complex I substrate; CI&IIP = phosphorylating respiration with complex I and II substrates; CI&IIE = maximum capacity of the electron transport chain with complex I and II substrates.

The FCRLI, FCRPI, and coupling efficiency significantly increased in F rats (Figure 5A,C,E), while the excess capacity was significantly lower (Figure 5F). In addition, the FCF was significantly higher for complex I, while the FCF for complex II was significantly lower (Figure 5G,H). All of the above changes were completely reversed in FR rats.

### 3.4. Markers of Oxidative Status

**Figure 6:**
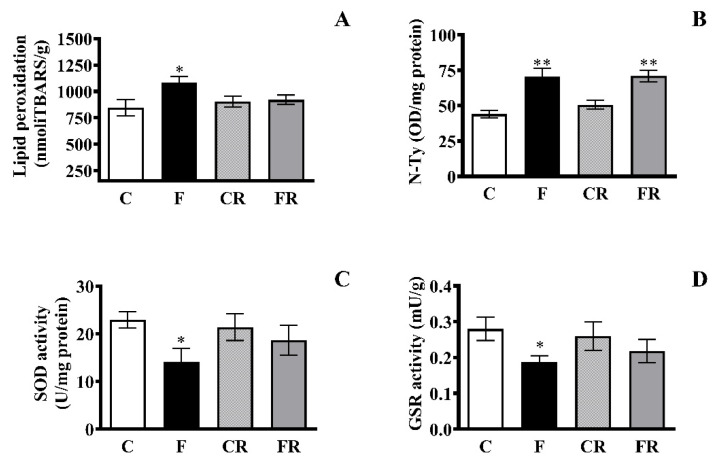
Markers of oxidative status. (**A**) TBARS (**B**) N-Tyr levels, (**C**) SOD activity, (**D**), GSR activity in the hippocampi of control (C), fructose-fed (F), control rescued (CR), and fructose-rescued (FR) rats. Data are the means ± SEM of eight rats/group. * *p* < 0.05 versus control rats; ** *p* < 0.01 versus control rats. Source of variation: one-way ANOVA followed by Tukey’s post-test.

As the strong link between mitochondrial function and reactive oxygen species (ROS) formation is well-known, we investigated the hippocampal oxidative status in the different rat groups and determined whether switching to a control diet can restore redox homeostasis alterations back to physiological levels. Lipid peroxidation was evaluated as a marker of oxidative damage to lipids, and N-Tyr, the footprint of protein oxidative damage induced by peroxynitrite [42], was assessed as a marker of oxidative damage to proteins. Further, the activity of two antioxidant enzymes, namely SOD, a frontline antioxidant enzyme catalyzing superoxide breakdown, and GSR, implicated in the recycling of reduced glutathione (GSH), was measured [43]. Enhanced diet-associated oxidative damage to lipids and proteins, namely increases in TBARS (*p* < 0.05; Figure 6A) and N-Tyr (*p* < 0.01; Figure 6B), were found in F rats. Of note, while the concentration of N-Tyr remained higher in FR with levels comparable to those found in hippocampi of F rats, the TBARS levels in FR rats returned to levels comparable to those in C rats. Likewise, we found diet-related decreases in SOD (Figure 6C) and GSR (Figure 6D) that were restored by switching back to the control diet.

### 3.5. Analysis of Neurofilament M and Synaptic Proteins

**Figure 7:**
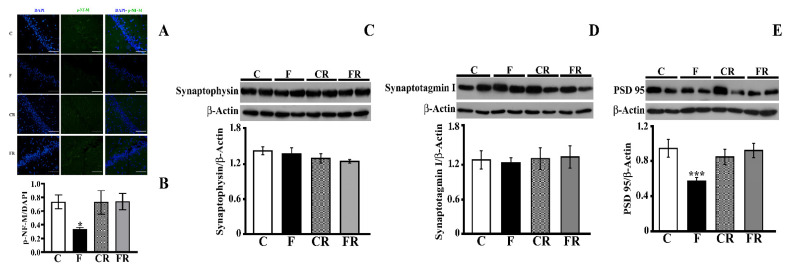
Markers of neuronal functioning. (**A,B**) p-Neurofilament M immunofluorescence (**C**) synapthophysin level (with representative Western blot), (**D**) synaptotagmin I (with representative Western blot), (**E**) PSD 95 level (with representative Western blot) in the hippocampi of control (C), fructose-fed (F), control rescued (CR), and fructose-rescued (FR) rats. Data are the means ± SEM of eight rats/group. * *p* < 0.05 versus control rats; *** *p* < 0.001 versus control rats. Source of variation: one-way ANOVA followed by Tukey post-test.

The impacts of both the fructose-rich diet and switching to a control diet on neurofilament M were then evaluated. Neurofilaments play crucial roles in axonal transport, and their alteration has been linked to the pathogenesis of neurological disorders involving cognitive dysfunction [44]. Therefore, to further study the effect of fructose on brain functioning, the level of p-NF-M in the hippocampus was determined by immunofluorescence analysis, and a significant decrease was found in F rats, but this was restored by the rescue treatment (Figure 7A,B).

Further, the levels of key pre (synaptophysin and synaptotagmin I) and post (PSD-95) synaptic proteins were measured in the hippocampi of all groups of rats (Figure 7C–E). Synaptophysin, the most abundant presynaptic vescicle protein, plays critical dual roles in both exocytosis and exoendocytosis coupling processes [45]. Synaptotagmin I, a major calcium sensor for transmitter release, participates in the clamping of synaptic vesicle fusion in mammalian neurons [46]. The fructose-rich diet did not affect the synaptophysin and synaptotagmin levels, while the concentration of PSD-95, which is involved in the function of neurotransmitter receptors, was significantly decreased in F rats (*p* < 0.001), but its levels returned to control values in FR rats.

## 4. Discussion

Changes in dietary lifestyle, such as the dramatic abuse of processed foods (bakeries, snacks, breakfast cereals) and bottled fruit juices rich in HFCS, particularly among young people, may have deleterious impacts on the body as well as on brain health by disrupting neuronal metabolism and function [7]. Since most fructose is known to be metabolized by the gut and liver before reaching the systemic circulation [47], a critical issue is determining how the intake of fructose can induce changes in brain structure and function. Indeed, although in small amounts, the fructose circulating in the blood can reach the brain, as demonstrated in vivo with mice that received an oral gavage of labeled fructose [47]. Therefore, it cannot be excluded that low concentrations of dietary fructose could have a direct impact on the brain. It is also possible that the systemic metabolism of fructose might promote the release of inflammatory cytokines and/or other plasma metabolites which, when imported into the brain, could affect its metabolism and function.

The present study deals with themes of primary importance that have not yet been deeply investigated: (i) the effect of short-term fructose intake on hippocampus function in the juvenile phase, which is critical for brain development and function, and (ii) the putative reversibility of hippocampus alterations induced by this sugar by switching to a control diet. In particular, in this study, we extended our previous results [16]. Firstly, we assessed the hippocampus levels of Glut-5, fructose, and uric acid and obtained further insight into the mitochondrial compartment by carrying out a full functional analysis of the oxidative phosphorylation system. In addition, we integrated information on the oxidative status by assessing antioxidant enzyme concentrations and deepened the analysis of the inflammatory status in response to fructose. Finally, to the best of our knowledge, this is the first investigation that has aimed to analyze the possible recovery from fructose-induced metabolic modifications in the hippocampus after a short-term dietary treatment.

Several studies have revealed that dietary fructose can increase brain expression of the fructose transporter Glut-5 [17,48] and sugar metabolism [49,50,51]. In this study, we showed that a short-term fructose-rich diet is associated with increases in both hippocampal Glut-5 and fructose levels. Interestingly, the increased levels of uric acid found in fructose-fed rats are suggestive of an enhancement in fructose metabolism in the hippocampus. In fact, in several tissues, it has been demonstrated that when fructose reaches the cells, fructokinase C converts it to fructose-1-phosphate with consequent decreases in intracellular phosphate and ATP levels. In turn, the low level of intracellular phosphate activates adenosine monophosphate (AMP) deaminase, with consequent degradation of AMP to inosine monophosphate and, eventually, uric acid. The consumption of AMP caused by the activation of AMP deaminase-2 reduces the cell’s ability to restore ATP levels and further stimulates uric acid production [8,52,53]. Uric acid, while being an anti-oxidant in the extracellular environment, has proinflammatory activity in the intracellular environment and can induce NF-kB activation and oxidative stress [54,55,56]. In particular, it has been reported that uric acid causes hippocampal inflammation via the TLR4/NF-kB pathway, resulting in cognitive dysfunction [56]. As a matter of fact, an increase in NF-kB activation was found in the hippocampi of fructose-fed rats together with an increase in the key inflammatory cytokine TNF-alpha as well as glial and microglial activation, evidenced by enhanced levels of both GFAP and Iba1. It is noteworthy that the diet-induced increases in fructose and uric acid levels occurred in parallel with the hippocampal inflammatory status, since the switch to a control diet normalized brain fructose and the uric acid level and brought back almost all the inflammatory parameters to values comparable to those of control rats. This result is different from that recently reported by Fierros-Campuzano et al. (2020) [18], who described the persistence of hippocampus inflammation markers, namely the increases of IL-1β and GFAP, in a group of rats exposed to a fructose-free period after fructose intake. Nevertheless, this difference could be ascribed to the much longer duration of the fructose diet (twelve weeks).

An intriguing finding of our analysis is the increase in Hpt in fructose-fed rats in the presence of no significant change in adiponectin. The Hpt increase persisted when the rats were switched back to a control diet for further 3 weeks. We previously showed that this acute-phase protein, which is well-known for its antioxidant activity [31,57,58,59], is highly sensitive to nutritional insults in the brain as well as in the systemic circulation [30,36,60], and its change, which persisted even after switching back to a control diet, might represent a protective mechanism against the enhanced oxidative stress found in the hippocampus. An interesting hypothesis that certainly deserves further investigation is that plasma Hpt, which is increased by fructose intake [61], might cross the blood–brain barrier by binding to specific receptors, or it might be produced locally in the brain following microglial activation and then characterized by a slow turnover. The latter hypothesis is supported by an investigation in which Hpt was found among the major selective transcripts expressed by microglia in the hippocampi of mice injected with a cocktail of cytokines (TNF-alpha, IL-12, and IL-1β) [62].

The onset of inflammation has been frequently associated with mitochondrial dysfunction and oxidative stress [43,63]. We, therefore, sought to investigate mitochondrial respiratory function by using, for the first time in hippocampi of young rats after fructose intake, the HRR on hippocampus homogenates to maintain mitochondria in a cellular context [24].

The decrease in ADP-supported respiration that was evident only after the addition of succinate is indicative of the fact that the impairment specifically affects the function of complex II. Lower ADP-supported respiration with complex I- and II-linked substrates may result from damage to complex II, complex III, complex IV, dicarboxylate carrier, and/or the phosphorylation reactions (Adenine Nucleotide Translocator, ATP synthase and phosphate carrier). The decreased respiration measured under uncoupled conditions allows us to exclude the occurrence of an impairment in phosphorylating reactions that do not exert control over respiration in this condition. In addition, the fact that a decrease in uncoupled respiration was also evident after the addition of rotenone, a specific inhibitor of the flux from complex I to complex II, thus allowing us to measure only the flux through the respiratory chain from complex II onwards, confirms that the fructose-induced impairment is located from complex II onwards. Similar to our findings, Agrawal et al. [64] found a decrease in hippocampal mitochondrial activity using succinate, a complex II-linked substrate after 7 weeks of consuming 15% fructose in drinking water. It has been suggested that complex II plays a role in reactive oxygen species (ROS) production under physiological and pathophysiological conditions, and defective functioning of complex II has been associated with neurodegeneration. In fact, the administration of an irreversible inhibitor of succinate dehydrogenase simulates the neuropathological and clinical features of Huntington disease (HD) in nonhuman primates [65] and evidence of the malfunctioning of complex II has been shown in patients with HD [66].

Moreover, the hippocampal mitochondria showed an increased coupling efficiency, which, in conjunction with the impairment in complex II, may contribute to the increased oxidative stress observed in F rats. In fact, uncoupling is a major mechanism in the control of mitochondrial ROS production, since it reduces the supply of electrons to the respiratory complexes and their possible interaction with oxygen [67,68]. The alteration in mitochondrial functioning is not linked to a lower organelle mass, since the hippocampus expression of PGC-1α was not altered in fructose-fed rats. The existing link between increased fructose delivery to the brain and the following mitochondrial impairment is supported by full reversal of the above changes in hippocampal mitochondria after switching back to the control diet.

ROS levels depend on the production of superoxide and its toxic metabolites as well as on the antioxidant defense mechanisms [43]. In line with the finding of mitochondria dysfunction, our results demonstrate that a fructose diet is associated with brain oxidative stress in terms of increased oxidative damage and decreased antioxidant defenses. N-Tyr and TBARS levels were significantly higher in the hippocampi of fructose-fed rats, and after switching to the control diet, TBARS returned to values comparable to those of control rats, while N-Tyr levels remained higher. This result can be explained by the fact that N-Tyr is a very stable marker of oxidative/nitrative stress [69,70] and suggests that protein turnover may control the return of the N-Tyr concentration to the initial values. As a matter of fact, brain protein turnover depends on multiple factors, such as the cell type, intracellular environment, specific protein functions, and protein interactions [71], with the half-lives of neuronal protein ranging from <2 to >14 days [71].

The unbalanced redox homeostasis is corroborated by the decrease in the activity of two antioxidant enzymes: SOD and GSR [43]. Consistent with the importance of SOD and GSR for cellular health, many human diseases of the central nervous system involve perturbations in these enzymes [72,73]. Regarding the analysis of these antioxidant enzymes, 3 weeks after the cessation of the fructose-rich diet, their activities returned to control values, suggesting that the fructose-induced alterations in redox homeostasis in young age can be reversed by a diet correction for an equal period of time.

To further highlight the key neuronal components influenced by fructose intake, we studied NF-M, as it is involved in the stabilization of newly‑sprouted axonal processes [74]. Neurofilaments guarantee the morphology of neurons and are crucial for axonal transport [74,75,76]. It is worth mentioning that disruption of the cytoskeletal framework of neurons typically triggers dystrophic neurites, thus representing a key feature of neurodegenerative diseases [77,78,79]. Based on the results of the immunofluorescence analysis, it can be suggested that alterations in NFs following fructose intake might give rise to dysfunction in axonal transport. This alteration seems to be reversible by switching to a control diet. A similar result was obtained by assessing the amount of synaptic proteins in the hippocampi of the rats in the different groups. In fact, a significant decrease in the post-synaptic critical protein PSD-95 was found in the hippocampi of fructose-fed rats, which was recovered by interrupting the fructose diet and switching to a control diet.

## 5. Conclusions

The picture that emerges from this study, which was conducted on a young rodent model, confirms that fructose can strongly impact brain function in juvenile age by promoting hippocampal inflammation, mitochondrial dysfunction, oxidative stress, alteration in cytoskeletal components, and post-synaptic proteins. These changes could undoubtedly have an important impact on neuronal activity and, in general, on cognitive function, especially in the youth, a very critical phase of brain development. Most of the alterations induced by a fructose-rich diet can be rescued by switching back to a control diet. Notable exceptions are represented by Hpt and N-Tyr, markers of inflammation and oxidative stress, respectively, which remain higher as an imprint of the previous damage. Investigation of the real consequences of the persistent alterations in these markers certainly deserves further attention and may represent an issue for further study. It cannot be excluded that a longer period of fructose intake could promote cerebral alterations to a greater extent that are difficult to revert with the return to a healthy diet. This study, once again, draws attention to the need to foresee the use of alternative sugars to HFCS with less dangerous effects to preserve the brain health of young populations.

## Figures and Tables

**Table 1:** Ingredients and nutritional composition of experimental diets.

Ingredients, g/100 g	Control Diet	Fructose Diet
Standard Chow ^a^	50.5	50.5
Sunflower Oil	1.5	1.5
Casein	9.2	9.2
Alphacel	9.8	9.8
Cornstarch	20.4	-
Fructose	-	20.4
Water	6.4	6.4
AIN-76 mineral mix	1.6	1.6
AIN-76 vitamin mix	0.4	0.4
Choline	0.1	0.1
Methionine	0.1	0.1
**Energy content and composition**		
Gross Energy Density (kJ/g)	17.2	17.2
ME content (kJ/g) ^b^	11.1	11.1
Proteins (% ME)	29.0	29.0
Lipids (% ME)	10.6	10.6
Carbohydrates (% ME)	60.4	60.4
Of which		
Fructose	-	30.0
Starch	52.8	22.8
Sugars	7.6	7.6

^a^ 4RF21, Mucedola, Italy; ^b^ Estimated by computation using values (kJ/g) for energy content as follows: proteins 16.736, lipids 37.656, and carbohydrates 16.736. ME = metabolizable energy.

## Data Availability

Data is contained within the article.
